# Impact of tongue base mucosectomy on quality-of-life outcomes: systematic review and single-centre experience

**DOI:** 10.1007/s00405-024-08976-4

**Published:** 2024-10-04

**Authors:** Daniel W. Scholfield, Andrew J. Williamson, Nina Cunning, Zaid Awad

**Affiliations:** 1 Department of Otolaryngology Head and Neck Surgery, Imperial College Healthcare, NHS Trust, London, W6 8RF UK; 2 Department of Surgery and Cancer, Imperial College London, London, UK

**Keywords:** Trans-oral robotic surgery, Tongue base mucosectomy, Occult primary, Unknown primary

## Abstract

**Purpose:**

Tongue base mucosectomy (TBM) is a well-established procedure in investigating cervical squamous cell carcinoma of occult primary. However, its risks have not been balanced against its benefits with validated tools.

**Methods:**

A systematic literature review was conducted for reported complications and quality-of-life outcomes following TBM. The complications and quality-of-life outcomes following TBM at our institution are then reported using objective metrics and validated assessment tools, including Performance Status Scale for Head and Neck Cancer Patients (PSS-HNS), University of Washington Quality-of-life Questionnaire (UW-QOL) and M. D. Anderson Dysphagia Inventory (MDADI).

**Results:**

Eighteen studies met the criteria for inclusion in the systematic review. Of these, 9 addressed swallowing outcomes described in text, without using validated assessment tools. No studies reported taste, speech and pain outcomes after TBM. Post-operative bleeding was not consistently reported. 20 patients underwent robotic TBM at our institution between 2017 and 2023. The primary tumour was identified in 50% (10/20) of cases. The median time to commencing soft diet and median time of NG feeding was 0 days. The median return to normalcy of diet score was 95. Median post-treatment UW-QOL pain and swallowing scores were 100 and 70 respectively. The median speech score was 100, saliva 70, and taste 70. The median normalised MDADI scores were: global 80; emotional 67; functional 80 and physical 65.

**Conclusions:**

Validated assessment tools better inform patients about treatment options and can help compare post-TBM results across institutions. Our data demonstrates that TBM patients have a functional post-operative swallow, are pain and gastrostomy free, even after adjuvant treatment. Routine post-operative insertion of NG tube is not necessary.

**Supplementary Information:**

The online version contains supplementary material available at 10.1007/s00405-024-08976-4.

## Introduction

Cervical squamous cell carcinomas of occult primary (SCCOP) accounts for 2–5% of all squamous cell carcinomas of the head and neck [1]. Most SCCOP originate in the oropharynx, especially if associated with Human Papilloma Virus (HPV). Modern practice includes thorough history, full examination including flexible nasophayngolaryngoscopy, cross-sectional imaging by computerised tomography (CT) and/or magnetic resonance imaging (MRI), and positron emission tomography-computed tomography (PET-CT) [2]. Examination under anaesthesia (EUA) of the upper aerodigestive tract (UADT) with directed biopsies and unilateral or bilateral diagnostic tonsillectomy is the next step [2]. PET CT identifies 50% of SCCOP which are negative in traditional cross-sectional imaging [3].

Tongue base mucosectomy via a transoral robotic (RTBM) or laser microsurgery (LTBM) approach is an additional strategy in the identification of occult primary if all the above is unsuccessful and is recommended by the United Kingdom National Guidelines for the investigation and management of neck SCCOP [2]. The National Institute for Health and Clinical Excellence (NICE) recommends that TBM is offered if facilities and expertise exist [4].

The value of RTBM has been demonstrated by a prospective multicentre cohort study of 32 SCCOP unidentified on PET CT [5]. In 17/32 (53%) of patients the primary tumour was identified in the tongue base using TBM. Transoral laser microsurgery has also been shown to be more effective than EUA and random biopsies in detecting the unidentified primary site [6].

**Fig. 1:**
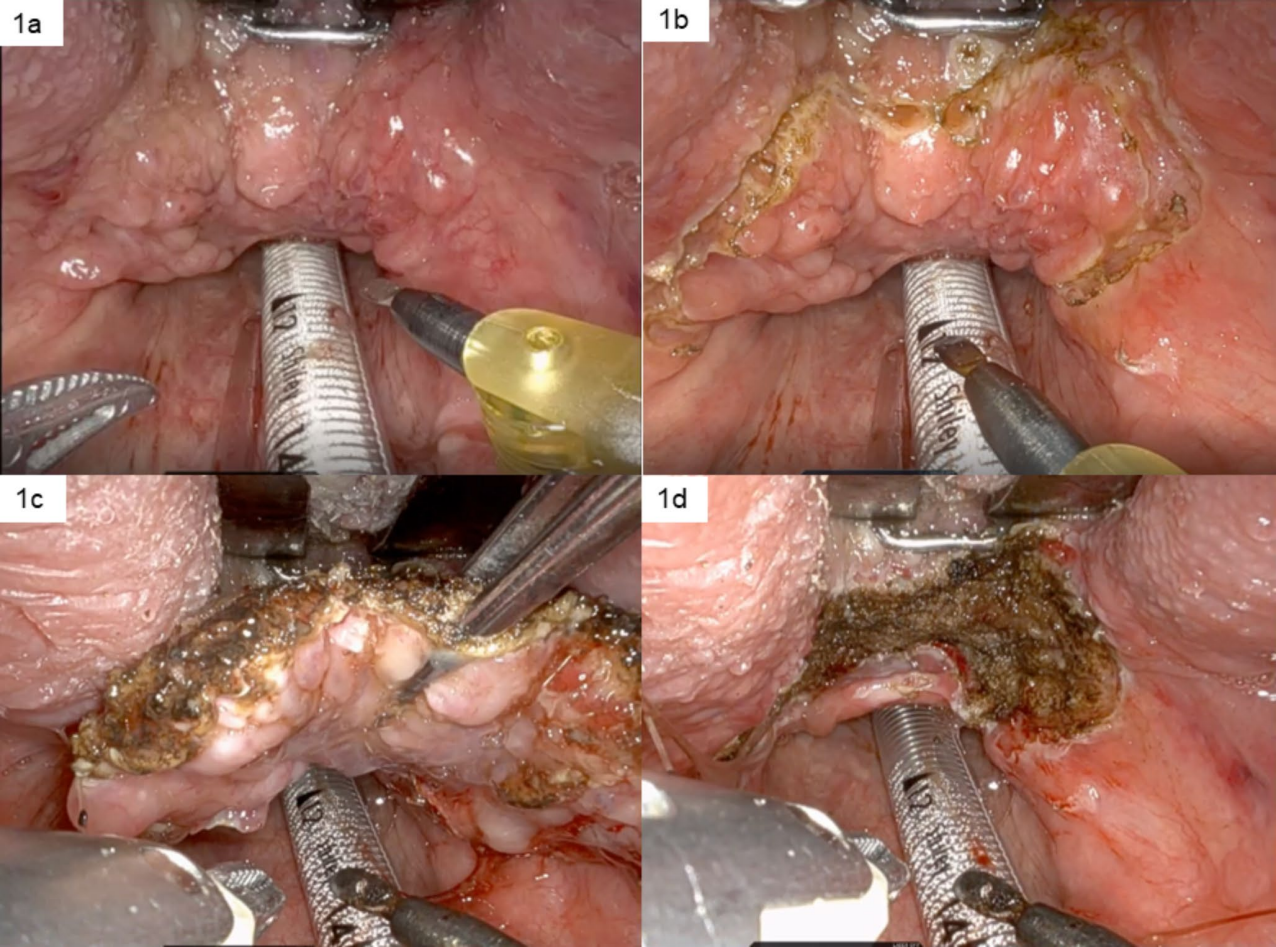
Robotic tongue base mucosectomy: **2a**: shows the view with the landmarks, **2b**: shows demarcation of the resection area, **2c**: shows orientation and removal of the specimen with the bedside assistant, and **2d**: shows the resection bed after removing the specimen

Tongue base mucosectomy involves en-bloc excision of the lymphoid tissue from the base of tongue, preserving the tongue musculature. The circumvallate papillae act as the anterior limit and the vallecula as the posterior limit of dissection. The lateral borders are the glosso-tonsillar sulci and the tonsils are often taken in addition to the tongue base if not previously excised. Transoral CO_2_ laser or robotic assisted surgery can be used as surgical expertise and equipment allow. At our institution the Da Vinci robot is used (Intuitive Surgical Inc., Sunnyvale, CA) which relies on monopolar diathermy for cutting. The advantages of using the robot include enhanced visualisation due to the use of a 30-degree high definition, three-dimensional endoscope which allows close binocular vision, and the manoeuvrability of the arms allow angulated approach addressing challenges posed by line of sight and mouth opening. This overcomes the challenge of TLM, where the operating field needs to be in an unobstructed line from the microscope with the target area being 400 mm away. Figure 1 demonstrates the improved view achievable with TORS and the stepwise approach. Accurate en-bloc dissection can reduce the likelihood of missing a small primary. TLM on the other hand, utilises the precision and haemostatic properties of the CO_2_ laser, without the charring effect or muscle contraction of monopolar diathermy on the resected specimen and surrounding tissue.

Identifying the primary tumour site can have a significant impact on planning radiotherapy fields and avoids total mucosal radiation (TMR) where the primary is not located. TMR to the UADT leads to significant dysphagia and xerostomia in the acute phase and at late follow-up [7]. A single-centre prospective phase II trial by Richards et al. (2016) assessed the complications of TMR with Intensity-modulated Radiotherapy (IMRT) for SCCOP in 36 patients. The incidence of severe (Grade 3) late dysphagia was 33%, mucositis was 42% and xerostomia was 64%. Of the patients with grade 3 dysphagia, 83% required feeding via nasogastric tube and 17% via gastrostomy tube [7]. Swallowing problems can result in greater morbidity, including malnutrition, dehydration and pneumonia [8], alongside prolonged hospital stay and ITU admissions. If TBM is successful in identifying the primary site and in some cases, potentially allows a margin-negative resection then targeted IMRT can reduce swallow dysfunction. Graboyes et al. (2014) managed 16% (9/57) of patients whose primary had been identified by RTBM or LTBM with surgery alone and no adverse pathological features after concurrent neck dissection. This treatment algorithm on a small patient cohort had a 97% disease-free survival with a median follow-up of 42 months [9].

With the above in mind, it is important to consider that no surgical procedure is free of risks. Post-operative haemorrhage is the predominant complication after TBM. A meta-analysis of transoral RTBM reported haemorrhage in 4.9% (19/387) of patients [10]. This risk of bleeding is similar to elective tonsillectomy, at 5.7–6.4% [11], but in SCCOP patients, bleeding can effect treatment timeline, further surgery and patient confidence, as well as being a life-threatening event.

Despite TBM being utilised to reduce the morbidity of radiotherapy, TBM itself can have an impact on swallowing as well as other risks such as loss of taste and post-operative haemorrhage. Dysphagia and post-operative pain are therefore important outcomes to balance against primary identification rates, in addition to reporting surgical complications.

## Materials and methods

### Systematic review

A systematic review of outcomes and complications of TBM was performed. MEDLINE and PubMed were searched from inception to March 2024. “Transoral robotic surgery,” “transoral laser surgery,” “tongue base” and “mucosectomy” were used as index terms.

**Fig. 2:**
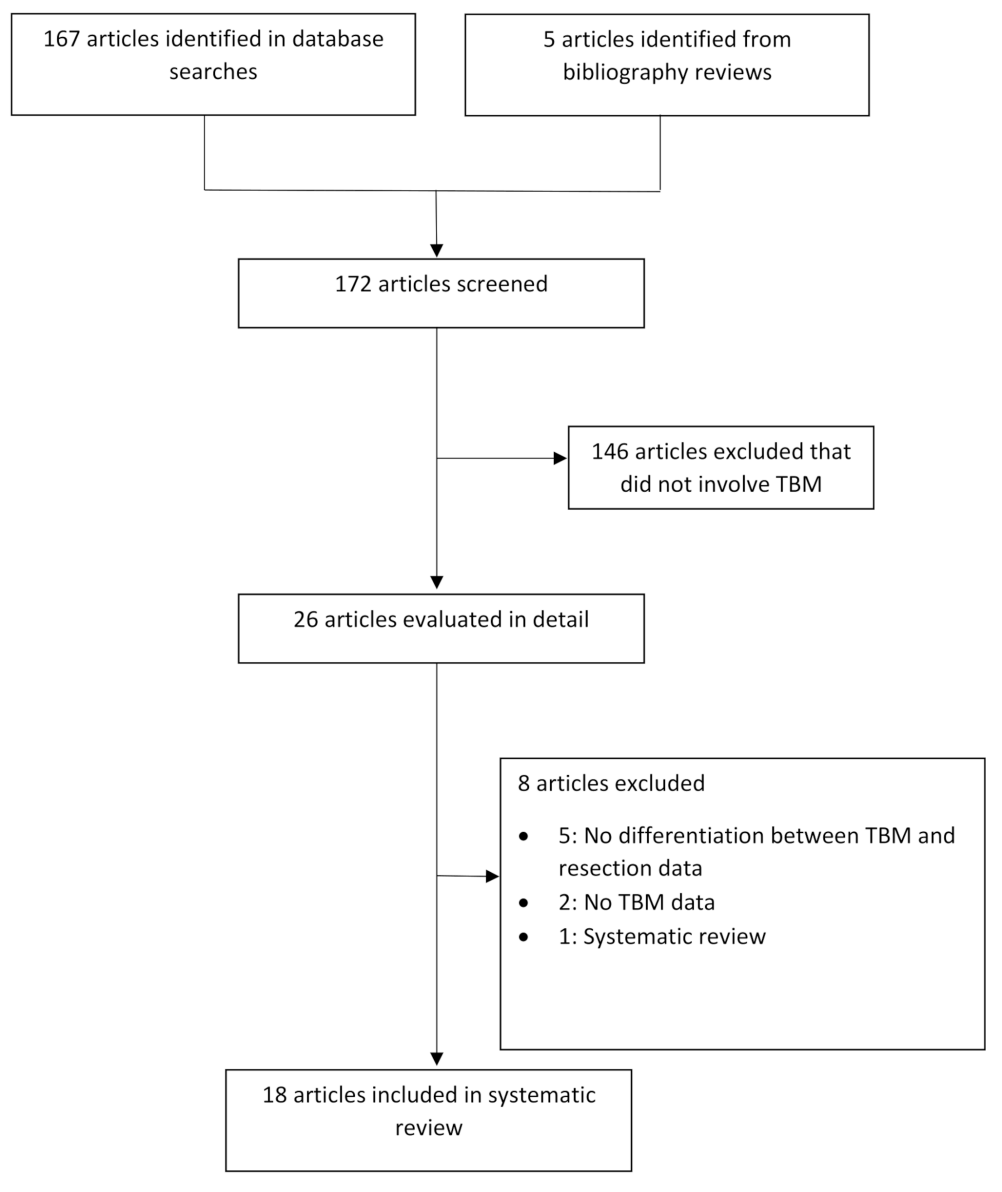
PRISMA flow diagram of study selection

Abstracts and titles of all identified studies were screened for further full text review. Non-English language and non-original studies (case reports, editorials, conference proceedings, reviews and meta-analyses) were excluded. Reference lists of the full articles were then manually searched for additional studies. The study selection process was documented using a PRISMA flow diagram (Fig. 2).

Data was extracted in accordance with the primary outcomes, namely post-operative pain and swallowing outcomes. Secondary data extracted included taste, primary aim of study, primary identification rate, gastrostomy tube insertion and complications including post-operative haemorrhage. Statistical analysis was performed using the meta and metafor packages in R studio v2023.12.1 + 402 (Boston, USA). A random effects meta-analysis was performed, of pooled proportions of primary site detection and post-operative haemorrhage. Forest plots were generated with a generalised linear mixed model and logit transformation, with the Clopper-Pearson method used to generate 95% confidence intervals. Heterogeneity was assessed using I^2^ statistic with *p* values < 0.05 considered significant.

### Institutional case series

Data was collected on 20 patients at our institution who underwent transoral robotic assisted TBM for the investigation of SCCOP from January 2017 – April 2023. Data collected included demographics, risk factors, HPV status, identification of the primary malignancy, final diagnosis, treatment, complications, enteral feeding and hospital stay. Quality-of-life and swallow outcomes are collected as part of routine follow-up at our institution. These include the Performance Status Scale for Head and Neck Cancer Patients (PSS-HNS) [Supplementary Material 1A], University of Washington Quality-of-life Questionnaire (UW-QOL) [Supplementary Material **1B**] and M. D. Anderson Dysphagia Inventory (MDADI) [Supplementary Material 1C]. MDADI scores were normalised to range from 20 (extremely low functioning) to 100 (high functioning). The University of Washington Quality-of-life Scale was developed and validated specifically for head and neck cancer [12]. It is simple to understand, multi-factorial and does not require input from clinicians. It consists of 12 questions that have between 3 and 6 response options that are scaled from 0 (worst) to 100 (best) according to the hierarchy of response. We retrospectively analysed the five most relevant to function after TBM (pain, swallowing, saliva, taste and speech).

## Results

### Systematic review

Eighteen studies met the inclusion criteria (Table 1) [5, 13–29] Nine studies addressed swallowing outcomes, the majority of which showed encouraging results. Hatten et al. (2017) reported 100% (60/60) patients had a return to normal swallow prior to discharge [14]. Another case-series reported 90% (9/10) of patients had returned to soft diet on their first post-operative visit, although one patient required gastrostomy feeding [5]. Mistry et al. (2020) described 96% (27/28) of patients to return to normal oral intake after 48 h, and 7% (2/28) to require gastrostomy feeding [25]. Krishnan et al. (2017) reported a mean time to normal swallowing function of 2.7 days [15]. However, Owen et al. (2017) reported comparably poor swallowing function in a prospective cohort study. The mean number of days of tube feeding was 14 and 33% (2/6) required this for more than 42 days [16]. Mettias et al. (2024) was the only study to report swallow function after the immediate post-operative period, reporting 38% (6/16) patients to be on a modified diet at 6 months post-operatively [28]. One study assessed short-term pain outcomes using the University of Washington Quality-of-life Survey. All 5 patients had little or no pain at first post-operative visit [19]. No studies reported TBM impact on taste.

**Table 1:** Summary of systematic review on tongue base mucosectomy

Author	Patient Number	TBM Surgical approach	Primary Objective	Quality of Life Outcomes				Complications		Primary detection rate (%)	Other primary objective outcomes
				Pain	Swallow	Gastrostomy	Voice	Haemorrhage % (*N*)	Other % (*N*)		
Kubik (2021) [13]	23	RTBM	Primary detection rate in HPV -ve SCC	N/A	N/A	N/A	N/A	4.3% (1/23)	0/23	13% (3/23) in HPV-ve SCC	-
Winter (2017) [5]	32	RTBM	Primary detection rate	N/A	N/A	N/A	N/A	5.7% (2/32)	1/32 HAP (3%)	53% (17/32)	-
Hatten (2017) [14]	60	RTBM	Primary detection rate	N/A	60 (100%) soft diet prior to discharge	N/A	N/A	5% (3/60)	N/A	80% (48/60)	-
Krishnan (2017) [15]	7	RTBM	Primary detection rate and complications	N/A	Average of 2.7 days before return to normal swallow function	N/A	N/A	0% (0/7)	N/A	71.4% (5/7)	-
Owen (2017) [16]	6	RTBM	Pre-treatment swallow to predict swallowing recovery	N/A	Average of 14 days tube feeding post-op	N/A	N/A	N/A	N/A	N/A	Poor pre-operative swallow correlated with post-operative duration of tube feeding
Channir (2015) [17]	13	RTBM	Primary detection rate	N/A	N/A	N/A	N/A	7% (1/14)	3/14 (21%)	54% (7/13)	-
Byrd (2014) [18]	22	RTBM	Cost effectiveness	N/A	N/A	N/A	N/A	0% (0/22)	2/22 (9%)	-	Incremental cost-effectiveness ratio of $8619 vs. EUA
Mehta (2013) [19]	10	RTBM	Primary detection rate	5/5 pain score of 75-100^a^	90% (9/10) soft diet at first post-op visit	10% (1/10)	N/A	0% (0/10)	N/A	90% (9/10)	-
Patel (2013) [20]	47	RTBM	Primary detection rate	N/A	N/A	N/A	N/A	8.5% (4/47)	N/A	72% (34/47)	-
Kuta (2018) [21]	27	LTBM	Efficacy of PET-CT TLM protocol	N/A	N/A	N/A	N/A	N/A	N/A	93% (25/27)	-
Nagel (2018) [22]	19	LTBM	Primary detection rate	N/A	N/A	N/A	N/A	5% (1/19)	N/A	75% (39/52)	-
Davies-Husband (2018) [23]	9	Endoscopic monopolar	Primary detection rate	N/A	All swallows normalised	N/A	N/A	0% (0/9)	N/A	44% (4/9)	-
Wallis (2018) [24]	12	Endoscopic monopolar	Primary detection rate	N/A	All returned to pre-op swallow	N/A	N/A	0% (0/12)	N/A	50% (6/12)	-
Mistry (2020) [25]	28	RTBM	Primary detection rate and complications	N/A	96% (27/28) return to normal oral intake 48 h	7% (2/28)	N/A	10% (3/28)	N/A	68% (19/28)	-
Olaleye (2022) [27]	12	RTBM	Feasibility study	N/A	N/A	N/A	N/A	N/A	N/A	42% (5/12)	Feasible robotic system
Olaleye (2023) [26]	14	LTBM	Feasibility study	N/A	93% (13/14) return to normal oral intake 24 h	7% (1/14)	N/A	7% (1/14)	N/A	50% (7/14)	Feasible LTBM system
Mettias (2024) [28]	16	RTBM	Primary detection rate, IDDSI, effect on pathway	N/A	75% (12/16) return to normal diet post-op. 38% (6/16) on modified diet at 6 months	0% (0/16)	N/A	0% (0/16)	1 readmission for pain	44% (7/16)	No detrimental effect on cancer pathway
Hardman (2024) [29]	58	RTBM / LTBM / endoscopic monopolar TBM	Outcomes of SSS histopathological technique	N/A	N/A	N/A	N/A	N/A	N/A	50% (29/58)	SSS has minimal additional diagnostic benefit with added histopathological workload.
Current study	20	RTBM	Functional outcome using UWQOL, MDADI	100^a^	70^a^ 80^b^	0% (0/20)	100^a^	5% (1/20)	4/20 (20%)	50% (10/20) primary detection rate	Functional post-operative swallow
**Total**								**5.1%** (17/333)		**62%** (274/440)	

IDDSI = International Dysphagia Diet Standardisation Initiative; SSS = step serial sectioning; UWQOL = University of Washington Quality of Life ; MDADI = MD Anderson Dysphagia Inventory; ^a^University of Washington Quality of Life; ^b^MDADI Global Score. N/A = Not reported

**Fig. 3:**
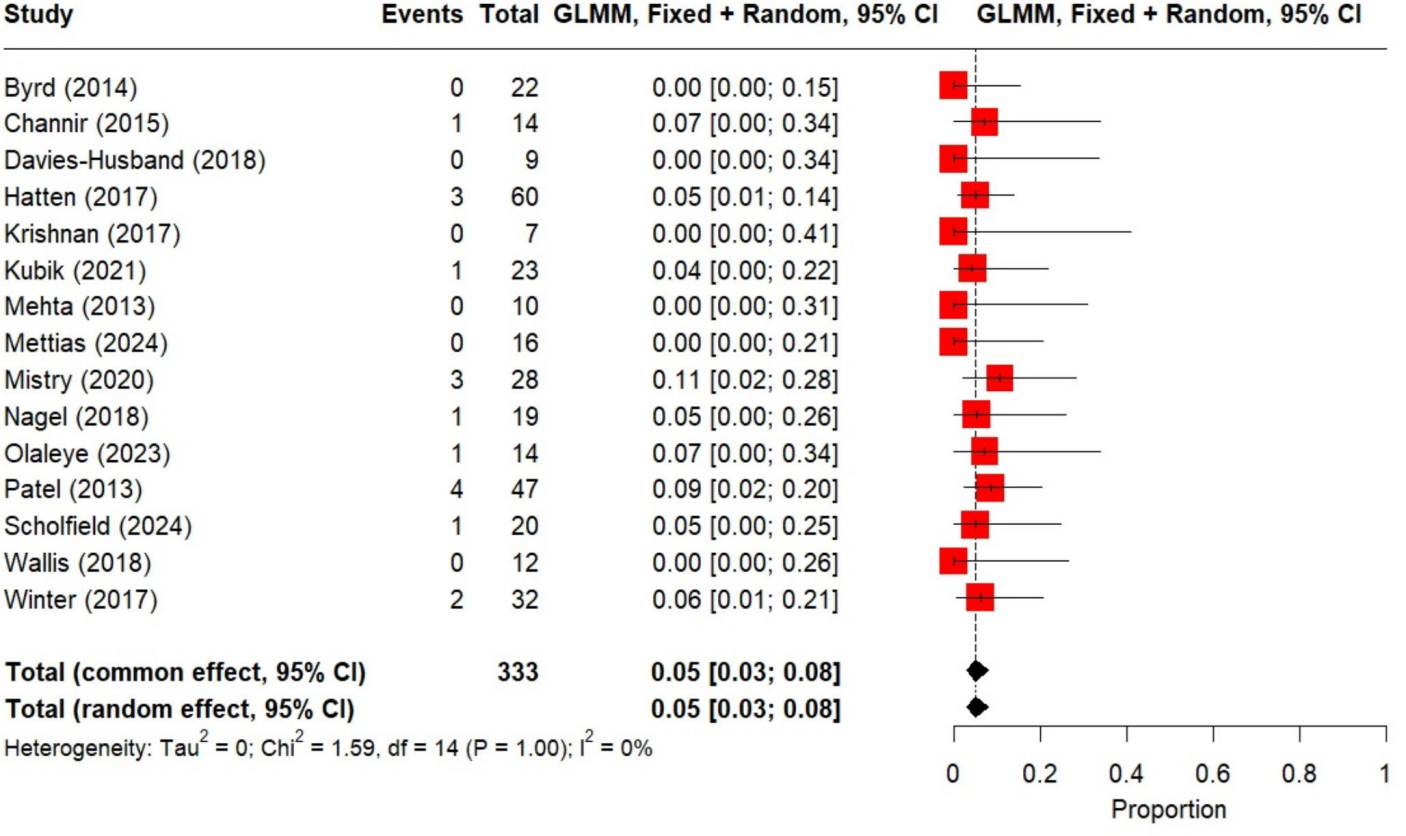
Forest plot showing random effects pooled post-operative haemorrhage rate across 15 studies

**Fig. 4:**
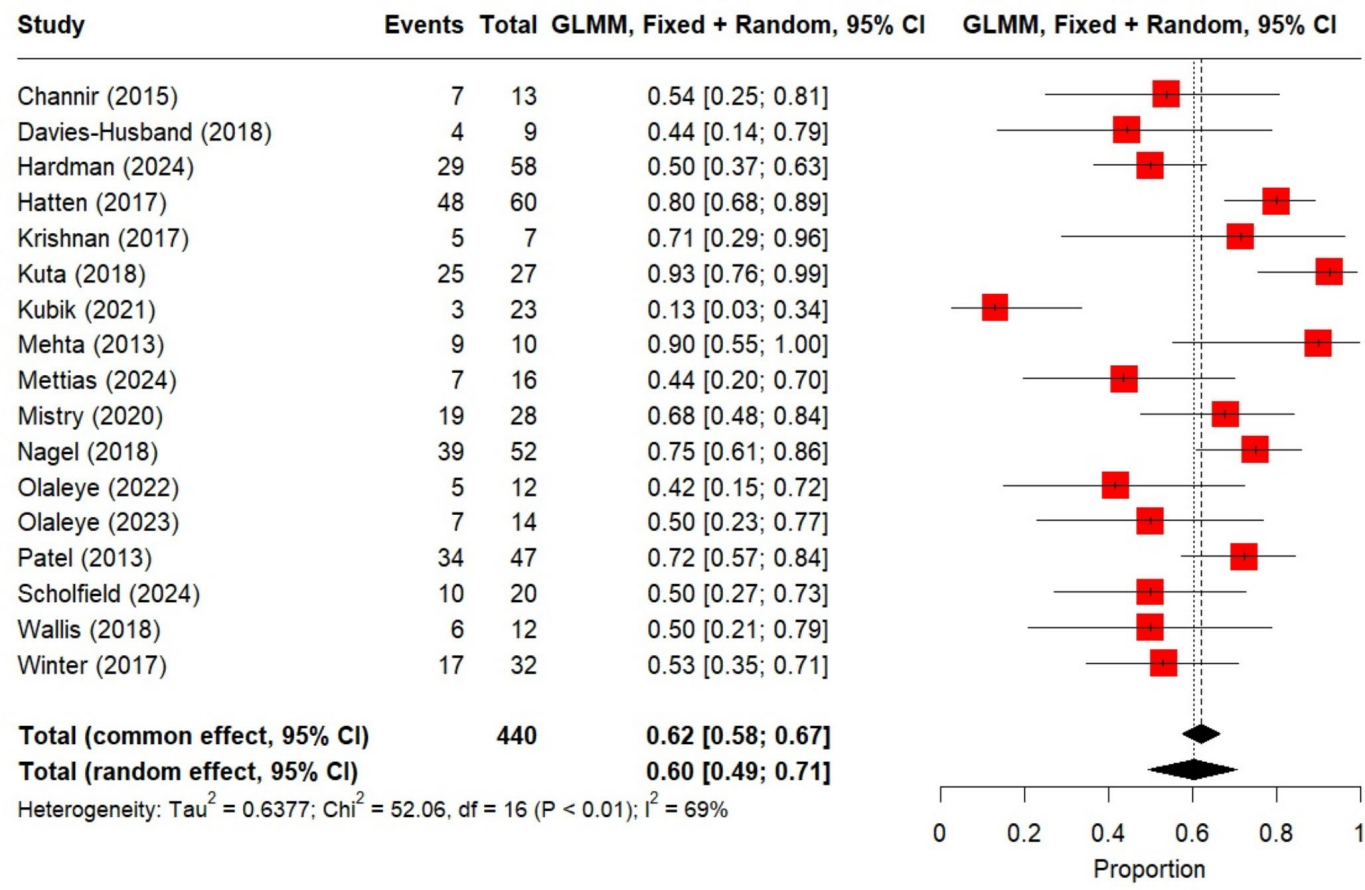
Forest plot showing random effects pooled primary site detection rate across 17 studies

Three studies did not address pain or swallow outcomes but included information on complications. Channir et al. (2015) reported short-term complications in 31% (4/13) of patients: one base of tongue bleed and neck haematoma requiring return to theatre, one temporarily impaired sensitivity of the tongue; one pulmonary embolus requiring ITU admission and one short-lasting severe pain, with no further details given [17]. Byrd et al. (2014) focused on cost effectiveness in 22 patients and reported one readmission for severe pain and dehydration requiring nasogastric tube feeding, 8 days after TBM [18]. Kubik et al. (2021) reported on TBM for HPV-negative CUP [13]. Post-operative haemorrhage rates were described in 15 studies containing 333 patients. The random effects pooled bleeding rate was 5% (95% CI 0.03: 0.08, I^2^ = 0%, *p* = 1.0; Fig. 3). No treatment associated mortality occurred. Primary site detection was reported in 17 studies totalling 440 patients. Random effects pooled rate of a positive TBM specimens was 60% (95% CI 0.49:0.71, I^2^ = 69%, *p* < 0.001; Fig. 4).

Most of the literature on TLM focusses on the primary resection of biopsy proven SCC. LTBM has been implemented in three studies that use a protocol of laser assisted biopsies followed by TBM if frozen sections are negative [21, 22, 26]. Kuta et al. (2018) do not report complications or pain and swallow outcomes for the 27 patients in their series [21]. Nagel et al. (2014) reported on 19 patients who had TLM lingual tonsillectomy after negative frozen section. One patient had a postoperative bleed that required return to theatre. All patients’ swallowing was evaluated post-operatively with the Functional Outcome Swallowing Score, a clinical measure of swallowing function. All patients had a score of 0 or 1, indicating normal physiologic swallowing [22]. Olaleye et al. (2023) similarly reported 93% (13/14) of patients to return to normal swallow after laser TBM and 7% gastrostomy rate [26].

Endoscopic TBM with hand-held monopolar diathermy was applied as an alternative to RTBM and LTBM [23, 24]. Both small case series reported no major complications and return to normal diet post-operatively.

### Case series

Twenty patients underwent TBM at our institution for SCCOP between January 2017 and April 2023, with a median age of 57 (range 39–70). Eight patients were smokers, four ex-smokers and seven patients had never smoked. Four patients’ alcohol intake was greater than 30 units/week. All cases were discussed at the multidisciplinary team (MDT) meeting with input from oncology, histology, radiology and nuclear medicine after confirmed SCC diagnosis from ultrasound-guided neck node core biopsy with HPV status. All patients had at least one form of cross-sectional imaging and PET CT.

P16 status in involved cervical lymph nodes was positive in 60% (12/20) of patients, however two of these had negative further HPV-DNA testing. The combinations of surgical intervention and adjuvant treatment are shown in Table 2. 12 patients had RTBM and tonsillectomy (11 bilateral; 1 unilateral) and 7 patients had RTBM alone due to previous tonsillectomy. 85% (17/20) of patient had neck dissections.

**Table 2:** Tumour and Treatment characteristics

Patient number	TNM AJCC 7th Edition	TNM AJCC 8th Edition	Location of primary	P16 status	HPV-DNA status	Surgery	Adjuvant treatment	Radiotherapy details	Chemotherapy details
1	T0N3M0	cTxN3bM0	Occult	Negative	NA	RTBM, bT	RT	65 Gy/30# macroscopic left neck and 54 Gy/30# ipsilateral uninvolved nodes	Nil
2	T0N2cM0	cTxN2cM0	Occult	Negative	NA	RTBM, bT	CRT	65 Gy/30# TMI and bilateral neck	Concomitant cisplatin
3	T1N2bM0	pT1N1M0	Tonsil	Positive	NA	RTBM, uT, uND	RT	Ipsilateral 65 Gy/30# to tonsillar bed and ipsilateral level II; 54 Gy/30# to lower neck	Nil
4	T2N2bM0	pT1N2M0	Tonsil	Positive	NA	RTBM, bND	RT	65 Gy/30# ipsilateral IMRT	Nil
5	T0N2bM0	pT0N1M0	Occult	Positive	NA	RTBM, uND*	CRT	60 Gy/30 IMRT TMI	Concomitant carboplatin
6	T0N2bM0	pT0N1M0	Occult	Positive	NA	RTBM, bT, uND	RT	Protons abroad	Nil
7	T1N2aM0	pT1N1M0	Tongue base	Positive	Positive	RTBM, bT, uND	RT	60 Gy/30 IMRT	Nil
8	T2N2bM0	T2N2bM0	Nasal septum	Positive	Negative	RTBM, bT, uND*	CRT	65 Gy/30 IMRT to nasal cavity, sinuses and bilat neck	Concomitant cisplatin and carboplatin
9	TxN1M0	TxN1M0	Occult	Negative	NA	RTBM, bT, uND*	RT	60 Gy/30# to parotid bed and right neck	Nil
10	T1N2bM0	T1N2bM0	Hypopharynx	Negative	NA	RTBM, bT, bND	CRT	65 Gy/30# to larynx, hypopharynx and bilat neck	Concomitant cisplatin
11	T1N1M0	pT1N1M0	Tongue Base	Positive	Positive	RTBM, uND	CRT	60 Gy/30# to oropharynx and bilateral neck	Concomitant cisplatin and carboplatin
12	T0N3aM0	TxN3aM0	Occult	Positive	Negative	RTBM, uND	RT	65 Gy/30# IMRT to potential primary sites and bilateral necks	Nil
13	T0N2aM0	TxN2aM0	Occult	Negative	Negative	RTBM, bT, uND	RT	60 Gy/30# IMRT TMI and bilateral neck	Nil
14	T0N2bM0	TxN2bM0	Occult	Negative	NA	RTBM, uND*	RT	60 Gy/54Gy in 30 fractions IMRT to right neck and potential primary sites	Nil
15	pT1pN2cM0	pT1pN3M0	Tongue base	Positive	Positive	RTBM, bT, uND	RT	60 Gy/30 to oropharynx and bilateral neck	Nil
16	pT2pN1M0	pT1N1M0	Tongue base	Positive	Positive	RTBM, bND*	RT	65 Gy/30# oropharynx and ipsilateral neck	Nil
17	T2N2bM0	T1N1M0	Tongue base	Positive	NA	RTBM, bT	CRT	65 Gy/30# oropharynx and bilateral neck	Concomitant cisplatin
18	T1N2bM0	T1N1M0	Tongue base	Positive	NA	RTBM, uND*	RT	60 Gy/30# oropharynx and ipsilateral neck	Nil
19	pT0N1M0	pTxN1M0	Occult	Negative	NA	RTBM, bT, uND*	Nil	Nil	Nil
20	T0N2bM0	TxN2bM0	Occult	Negative	Negative	RTBM, uND	Nil	Nil	Nil

RTBM = robotic tongue base mucosectomy; bT = bilateral tonsillectomy; uT = unilateral tonsillectomy; uND = unilateral neck dissection; bND = bilateral neck dissection; * = staged; RT = radiotherapy; CRT = chemoradiotherapy; IMRT = Intensity-modulated radiation therapy; TMI = total mucosal irradiation

The included cases had previously undergone careful examination of the upper aerodigestive tract under general anaesthesia with or without diagnostic tonsillectomy, prior to considering TBM. Table 3 summarizes the tumour and treatment characteristics of the whole cohort. Primary malignancy was identified in 50% (10/20) of patients: 2 patients had SCC of the tonsil, 6 of the tongue base, 1 of the nasal septum and 1 of the hypopharynx. The primary detection rate for HPV-positive disease was 80% (8/10), compared to 20% (2/10) for HPV-negative disease. 50% (10/20) therefore had a final diagnosis of HNSCC of unknown primary and underwent oncological treatment after MDT discussion with consideration of HPV status. 35% (7/20) had extra-capsular spread. The spectrum of adjuvant treatment combinations is shown in Table 2. Six patients (30%) had chemo-radiotherapy and 12 patients (60%) had radiotherapy alone. Two patients (10%) had no adjuvant treatment due to patient choice. Whenever possible, surgical procedures were combined to reduce the number of hospital admissions, general anaesthetics and meet treatment targets.

**Table 3:** Tumour and Treatment characteristics for the whole cohort

Characteristic	*N* (%)
**T Stage (AJCC 8th Edition)**	
0/Tx 1 2	10 (50%) 9 (45%) 1 (5%)
*N* Stage (AJCC 8th Edition)	
1 2 2a 2b 2c 3 3a 3b	10 (50%) 1 (5%) 1 (5%) 4 (20%) 1 (5%) 1 (5%) 1 (5%) 1 (5%)
**M Stage (AJCC 8th Edition)**	
0	20 (100%)
**Location of primary**	
Occult Tongue base Tonsil Nasal septum Hypopharynx	10 (50%) 6 (30%) 2 (10%) 1 (5%) 1 (5%
**P16 Status**	
Positive Negative	12 (60%) 8 (40%)
**HPV DNA Status**	
Positive Negative Not available	4 (20%) 4 (20%) 12 (60%)
**Surgery**	
Robotic tongue base mucosectomy Bilateral tonsillectomy Unilateral tonsillectomy Bilateral neck dissection Unilateral neck dissection	20 (100%) 11 (55%) 1 (5%) 3 (15%) 14 (70%)
**Adjuvant treatment**	
Radiotherapy Chemoradiotherapy None	12 (60%) 6 (30%) 2 (10%)

Median hospital stay was 1.5 days (interquartile range IQR 0-6.5) and all patients were gastrostomy free at most recent follow-up. Five patients had post-operative complications. One had tongue-swelling in recovery that required re-intubation, followed by extubation four days post-operatively. One patient (1/20; 5%) had a spontaneously resolving tongue base bleed that did not require return to theatre and one patient had a neck haematoma in recovery and was taken back to the operating room. Two patients had minor complications: one had epistaxis after nasal intubation that required packing and one had a post-operative urinary tract infection.

Median time of MDADI and UW-QOL data collection was 24.5 months post completion of treatment. Median PSS-HN data collection was 32 months. All patients had normal pre-operative swallow assessed by a specialist speech and language therapist. The median time to soft diet post-operatively was 0 days (IQR 0–1) and median length of NG feeding was 0 days (IQR 0–0). 90% (18/20) of patients had PSS-HN scores, taken at a median of 32 months after completion of treatment. The median understandability of speech score was 100 (IQR 100–100), equating to “always understandable.” The median return to normalcy of diet score was 95 (IQR 90–100), meaning a full diet with liquid assist, to full diet with no restrictions. The median grade of dysphonia was 0 (IQR 0–0), equating to no voice disorder.

UW-QOL data was collected for 18 patients (90%), at a median of 24.5 months (IQR 12.75–40.75 months) post-operatively. Median UW-QOL pain and swallowing scores were 100 (IQR 81.25–100) and 70 (IQR 70-86.25) respectively. The median saliva score was 70 (IQR 30–70), speech 100 (IQR 100–100) and taste 70 (IQR 70–100).

Complete MDADI data was collected for 18 patients (90%), at a median of 24.5 months post-operatively (IQR 15.5-40.75). The median global normalised score was 80 (IQR 65–100). The median normalised emotional score was 67 (IQR 60–82), functional score was 80 (IQR 69–88) and physical score was 65 (IQR 58–75).

When comparing patients who had CRT to patients who had RT alone, there were no noticeable differences in MDADI or UW-QOL scores across any domains.

## Discussion

TBM improves the detection rate of occult malignancy and has been shown to half the number of true SCCOP after PET CT and EUA UADT with tonsillectomy [5]. Its inclusion as a diagnostic technique in national guidelines reflects its efficacy and indicates its use will continue to increase [4]. However, TBM has its own challenges and comes with potential complications. The need to identify a primary is driven by the morbidity of radical treatment. Extensive irradiation of both sides of the neck and pharyngeal mucosa with concurrent chemotherapy results in significant xerostomia and dysphagia. The rate of dilatation for oesophageal stricture after TMR is 40% [30]. However, there is limited data on morbidity caused by TBM, which is implemented to target adjuvant radiotherapy . Previous systematic reviews do not explore functional outcomes after TBM [10, 31].

Saliva production is a problematic area for patients post head and neck cancer treatment. Xerostomia is a widely reported side-effect of radiotherapy and it has been shown to be the most important domain to patients in the UW-QOL score, with 33% choosing this side-effect as most problematic [32–34]. In our patient cohort saliva production had the lowest median UW-QOL score of 70, a figure that correlates with “less saliva than normal, but enough.”. There were no reports of dysphonia by patients in the UW-QOL score, or by speech and language therapist assessments. Median post-operative pain score was 100 at 24.5 months post-treatment. The lack of pain immediately post-operatively is demonstrated by a return to soft diet within 24 h, optimised by our local protocol which provides patients with regular opioid-based analgesia. Mehta et al. (2013) reported similar findings, with all five of their TBM patients reporting UW-QOL pain scores of 75–100 [19]. Pain management should be a priority in order to optimise swallow rehabilitation. Using topical local anaesthetic and consulting a pain specialist both help, the latter may adjunctively treat with neuropathic agents.

Dysphagia is a common effect of radiotherapy and the median UW-QOL swallowing score after completion of oncological treatment in our case-series was 70. This is lower than reported outcomes for oncological treatment alone which range between 80 and 84 [11, 35]. However, the median normalcy of diet score shows that TBM patients can expect to manage full diet with or without liquid assist after completion of treatment. Follow-up by the speech and language team is essential in rehabilitating swallow and monitoring improvement. The median MDADI emotional score in our series was 67. This is an aspect of swallow function that can often be overlooked and it is therefore important to address this with psychological support if necessary. The functional score reflects ability to perform daily activities and had a median score of 80 while the median physical score of 65 reflects the action of swallowing, weight maintenance and aspiration.

The global MDADI score consists of only one statement: “My swallowing ability limits my day-to-day activities,” which in this series gave a median global score of 80 (“Disagree”). This finding can be used to counsel patients, that change in swallow is unlikely to impact their day-to-day activities after TBM and curative treatment, along with the finding that all patients were gastrostomy free at latest follow-up. Contrary to the differences in UW-QOL swallowing score previously discussed, the global MDADI score compares favourably to chemoradiotherapy and radiotherapy in the literature – which gives a mean global MDADI score at 3 months of 61.3 for chemoradiotherapy, 66.3 for radiotherapy 63 Gy in 30 fractions and 76.8 for radiotherapy 50 Gy in 16 fractions [32]. This further supports the argument for TBM in potentially reducing the volume and therefore the total dose of radiation.

Owen et al. (2017) assessed whether pre-treatment swallowing measures predict swallowing recovery at 6 weeks after trans-oral robotic TBM [16]. The patients had a mean of 14 days of post-operative tube feeding, similar to transoral robotic assisted tongue base tumour resections (19 days). The mean length of tube feeding was likely impacted by the small patient numbers and early experience in the evolution of RTBM. The authors concluded that poor pre-operative swallow correlated with post-operative duration of tube feeding. This emphasises the need for formal swallow assessment and documentation pre-operatively, so patients that are more likely to have poor swallow outcomes post TBM can be identified and start “pre-habilitation”. That said, without extensive neck nodal disease, it is unlikely that those without an identifiable primary will have a poor swallow. Comorbidities should be considered when identifying high risk patients. For instance, chronic obstructive pulmonary disease can increase the likelihood of a gastrostomy tube need, as these patients are more vulnerable to aspiration pneumonia. The effects of age and neuromuscular disorders on swallowing function and recovery is not clearly demonstrated in the literature but should be considered in pre-operative work-up.

Our case series shows that the majority of patients can return to a soft diet within 12 h of TBM, with or without tonsillectomy and neck dissection. Three patients in our case-series had a delay in return to soft diet over 4 days. One did not return to soft diet for 9 days due to post-operative pain and UTI-related delirium. His oral intake was supplemented by nasogastric feed for a further 6 days. The second patient did not return to soft diet until 7 days post-operatively, due to poor initial pain control. The third patient did not return to soft diet before commencing radiotherapy – he had a gastrostomy tube inserted prophylactically, which was removed after completing treatment. His swallow was likely impacted by the need for early re-intubation for tongue swelling and intensive care admission for 4 days. At latest follow-up 41 months post-treatment, he had a functional MDADI score of 68 and normalcy of diet score of 50, correlating with soft chewable foods. An uneventful return to oral intake for the other 85% (17/20) of patients resulted in a median length of nasogastric feeding of 0 days. It has therefore become our institution’s practice to not routinely insert a nasogastric tube after TBM.

As illustrated in Table 1, previous studies have not addressed taste or speech outcomes and only one study has addressed pain outcomes after robotic TBM. Swallowing outcomes were described in 50% (9/18) of TBM studies. Those that addressed swallowing outcomes did not use validated head and neck specific assessments tools of dysphagia, such as MDADI and only one study reported beyond swallow function beyond the immediate post-operative period [28]. Those that reported on swallow outcomes showed rapid return to normal swallow [5, 14, 15, 25], apart from Owen et al. (2017) as previously mentioned [16]. The use of laser or endoscopic monopolar to undertake TBM also reported return to normal swallow [22–24, 26]. Hardman et al. (2024) reported on the outcomes of step serial sectioning histopathology from patients included in a prospective multicentre study on TBM. They are gathering MDADI and pain scores on these patients, but the functional outcomes are yet to be reported [29, 36]. The authors hypothesize a return to near normal swallow function occurs 6 weeks post-surgery, and subsequent swallow deterioration to be due to adjuvant radiotherapy [36].

Post-operative haemorrhage rates are similar in most studies to those after tonsillectomy [37]. It results in readmission and in some cases a return to theatre. As a life-threatening event, this should be reported consistently. The random effects pooled bleeding rate is 5% (95% CI 0.03: 0.08, I^2^ = 0%, = 1.0).

The majority of patients in our study had a bilateral tonsillectomy at the time of TBM with the aim of improving the primary identification rate. In the literature, there is currently no consensus as to whether unilateral or bilateral tonsillectomy is the optimum approach. We identified no malignancy in the contralateral tonsil, which reflects the low rate of contralateral tonsil malignancy in the literature: Farooq et al. (2019) described a contralateral tonsil malignancy rate of 0.9% [10] and Hardman et al. (2024) identified no malignancy in the contralateral tonsil [29]. The most recent United Kingdom guidelines state that at least unilateral tonsillectomy should be undertaken alongside TBM, and that contralateral tonsillectomy should be considered [2]. More data is required to identify if swallow function differs after unilateral or bilateral tonsillectomy alongside TBM and the benefit of a slightly increased detection rate should be weighed against the potential additional swallow morbidity of bilateral tonsillectomy [2].

Swallow, saliva production and pain are the three most important issues reported by head and neck cancer patients in their treatment [38]. The anticipated increase in TBM procedures indicate a need for consistent reporting of morbidity and studies with larger cohorts to quantify these outcomes after diagnostic TBM. Our case series is the first to employ a validated scoring method to assess swallow, pain, speech, taste and saliva production after TBM beyond the immediate post-operative period. Looking forward, a multi-centre database is required to record prospective functional and oncological outcomes of TBM patients.

### Limitations

Limitations of this study include the number of patients in our cohort, although this seems to be similar to other studies. The follow-up is variable in our study and the literature. Our study shows the variation in post-TBM oncological treatment, such as radiotherapy, chemoradiotherapy or nothing, and surgical procedures such as tonsillectomy and neck dissection concurrently or consequently. This makes it difficult to ascertain the impact of each modality on quality-of-life outcomes. The variation or lack of standardized measures used for quality-of-life outcomes precludes comparison of many functional aspects. The focus in the literature has been on oncological outcomes, rather than quality-of-life data. What constitutes a TBM and variation in resection samples may also vary across different centres.

## Conclusion

The investigation and management of SCCOP is a challenge that continues to evolve. PET-CT and now TBM, have increased the rate of identifying the primary. TBM is a relatively safe procedure with comparable rates of post-operative haemorrhage to elective tonsillectomy. The benefit of TBM is the avoidance of extensive mucosal radiation, which has a negative impact on quality-of-life. However, this must be balanced against the risks of TBM, which include immediate post-operative complications and impact on quality-of-life. Patients should be informed about the risks of TBM along with the potential benefits. Clinicians are encouraged to use validated tools to assess quality-of-life parameters along the patient pathway and an international standardized databank would be of great value. Our data demonstrates that RTBM does not add toxicity to chemoradiotherapy, and that patients have a functional swallow, are pain and gastrostomy free 24 months post-treatment. Routine insertion of post-operative NG tube is not necessary.

## Electronic supplementary material

Below is the link to the electronic supplementary material.

Supplementary Material 1

## Data Availability

Anonymised data available upon request.
