# Supplementary material for: Impact of tongue base mucosectomy on quality-of-life outcomes: systematic review and single-centre experience
Source: Eur Arch Otorhinolaryngol. 2024 Oct 4;282(2):1027–40. doi: 10.1007/s00405-024-08976-4 (PMC11805862; doi:10.1007/s00405-024-08976-4)
Supplement: Supplementary file 1 — Supplementary Material 1 [file 405_2024_8976_MOESM1_ESM.docx]

**Supplementary Material 1A**

**Performance Status Scale for Head and Neck Cancer Patients**

**Normalcy of Diet**

100 Full diet (no restrictions)

90 Full diet (liquid assist)

80 All meat

70 Raw carrots, celery

60 Dry bread and crackers

50 Soft chewable foods

40 Soft foods requiring no chewing

30 Pureed foods

20 Warm liquids

10 Cold liquids

0 Non-oral feeding (tube fed)

**Public Eating**

100 No restriction of place, food or companion

75 No restriction of place, but restricts diet when in public

50 Eats only in presence of selected persons in selected places

25 Eats only at home in presence of selected persons

0 Always eats alone

**Understandability of Speech**

100 Always understandable

75 Understandable most of the time; occasional repetition necessary

50 Usually understandable; face-to-face contact necessary

25 Difficult to understand

0 Never understandable; may use written communication

**Supplementary Material 1B:**

**University of Washington Quality of Life Questionnaire (UW-QOL)**

This questionnaire asks about your health and quality of life **over the past seven days**. Please answer all of the questions by ticking one box for each question.

1. **Pain**.

- I have no pain (100)
- There is mild pain not needing medication (75)
- I have moderate pain - requires regular medication (e.g. paracetamol) (50)
- I have severe pain controlled only by prescription medicine (e.g. morphine) (25)
- I have severe pain, not controlled by medication (0)

2. **Appearance**.

- There is no change in my appearance (100)
- The change in my appearance is minor (75)
- My appearance bothers me but I remain active (50)
- I feel significantly disfigured and limit my activities due to my appearance (25)
- I cannot be with people due to my appearance (0)

3. **Activity**.

- I am as active as I have ever been (100)
- There are times when I can't keep up my old pace, but not often (75)
- I am often tired and have slowed down my activities although I still get out (50)
- I don't go out because I don't have the strength (25)
- I am usually in bed or chair and don't leave home (0)

4. **Recreation**.

- There are no limitations to recreation at home or away from home (100)
- There are a few things I can't do but I still get out and enjoy life (75)
- There are many times when I wish I could get out more, but I'm not up to it (50)
- There are severe limitations to what I can do, mostly I stay at home and watch TV (25)
- I can't do anything enjoyable (0)

5. **Swallowing**.

- I can swallow as well as ever (100)
- I cannot swallow certain solid foods (70)
- I can only swallow liquid food (30)
- I cannot swallow because it "goes down the wrong way" and chokes me (0)

6. **Chewing**.

- I can chew as well as ever (100)
- I can eat soft solids but cannot chew some foods (50)
- I cannot even chew soft solids (0)

7. **Speech**.

- My speech is the same as always. (100)
- I have difficulty saying some words but I can be understood over the phone (70)
- Only my family and friends can understand me (30)
- I cannot be understood (0)

8. **Shoulder**.

- I have no problem with my shoulder (100)
- My shoulder is stiff but it has not affected my activity or strength (70)
- Pain or weakness in my shoulder has caused me to change my work / hobbies (30)
- I cannot work or do my hobbies due to problems with my shoulder (0)

9. **Taste**.

- I can taste food normally (100)
- I can taste most foods normally (70)
- I can taste some foods (30)
- I cannot taste any foods (0)

10. **Saliva.**

- My saliva is of normal consistency (100)
- I have less saliva than normal, but it is enough (70)
- I have too little saliva (30)
- I have no saliva (0)

11. **Mood.**

- My mood is excellent and unaffected by my cancer (100)
- My mood is generally good and only occasionally affected by my cancer (75)
- I am neither in a good mood nor depressed about my cancer (50)
- I am somewhat depressed about my cancer (25)
- I am extremely depressed about my cancer (0)

12. **Anxiety.**

- I am not anxious about my cancer (100)
- I am a little anxious about my cancer (70)
- I am anxious about my cancer (30)
- I am very anxious about my cancer (0)

**Supplementary Material 1C:**

**The M.D. Anderson Dysphagia Inventory**

My swallowing ability limits my day-to-day activities.

Strongly Agree Agree No Opinion Disagree Strongly Disagree

E2. I am embarrassed by my eating habits.

Strongly Agree Agree No Opinion Disagree Strongly Disagree

F1. People have difficulty cooking for me.

Strongly Agree Agree No Opinion Disagree Strongly Disagree

P2. Swallowing is more difficult at the end of the day.

Strongly Agree Agree No Opinion Disagree Strongly Disagree

E7. I do not feel self-conscious when I eat.

Strongly Agree Agree No Opinion Disagree Strongly Disagree

E4. I am upset by my swallowing problem.

Strongly Agree Agree No Opinion Disagree Strongly Disagree

P6. Swallowing takes great effort.

Strongly Agree Agree No Opinion Disagree Strongly Disagree

E5. I do not go out because of my swallowing problem.

Strongly Agree Agree No Opinion Disagree Strongly Disagree

F5. My swallowing difficulty has caused me to lose income.

Strongly Agree Agree No Opinion Disagree Strongly Disagree

P7. It takes me longer to eat because of my swallowing problem.

Strongly Agree Agree No Opinion Disagree Strongly Disagree

P3. People ask me, “Why can't you eat that?”

Strongly Agree Agree No Opinion Disagree Strongly Disagree

E3. Other people are irritated by my eating problem.

Strongly Agree Agree No Opinion Disagree Strongly Disagree

P8. I cough when I try to drink liquids.

Strongly Agree Agree No Opinion Disagree Strongly Disagree

F3. My swallowing problems limit my social and personal life.

Strongly Agree Agree No Opinion Disagree Strongly Disagree

F2. I feel free to go out to eat with my friends, neighbors, and relatives.

Strongly Agree Agree No Opinion Disagree Strongly Disagree

P5. I limit my food intake because of my swallowing difficulty.

Strongly Agree Agree No Opinion Disagree Strongly Disagree

P1. I cannot maintain my weight because of my swallowing problems.

Strongly Agree Agree No Opinion Disagree Strongly Disagree

E6. I have low self-esteem because of my swallowing problems.

Strongly Agree Agree No Opinion Disagree Strongly Disagree

P4. I feel that I am swallowing a huge amount of food.

Strongly Agree Agree No Opinion Disagree Strongly Disagree

F4. I feel excluded because of my eating habits.

Strongly Agree Agree No Opinion Disagree Strongly Disagree
